# Time Is Life: Golden Ten Minutes on Scene–EuReCa_Serbia 2014–2023

**DOI:** 10.3390/medicina60040624

**Published:** 2024-04-11

**Authors:** Suzana Randjelovic, Srdjan Nikolovski, Dragica Selakovic, Miodrag Sreckovic, Sara Rosic, Gvozden Rosic, Violetta Raffay

**Affiliations:** 1 Department of Emergency Medicine, University Clinical Center Kragujevac, 34000 Kragujevac, Serbia; suzanarandjelovic25@gmail.com; 2 Health Sciences Campus, Loyola University Chicago, Maywood, IL 60153, USA; 3 Faculty of Medicine, University of Belgrade, 11000 Belgrade, Serbia; 4 Department of Physiology, Faculty of Medical Sciences, University of Kragujevac, 34000 Kragujevac, Serbia; dragica984@gmail.com (D.S.); grosic@medf.kg.ac.rs (G.R.); 5 Department of Internal Medicine, Faculty of Medical Sciences, University of Kragujevac, 34000 Kragujevac, Serbia; sreckovic7@gmail.com; 6 Clinic of Cardiology, University Clinical Center Kragujevac, 34000 Kragujevac, Serbia; 7 Department of Medicine, School of Medicine, European University Cyprus, 2404 Nicosia, Cyprus; v.raffay@euc.ac.cy

**Keywords:** out-of-hospital cardiac arrest, cardiopulmonary resuscitation, european registry of cardiac arrest, time on scene, return of spontaneous circulation, survival

## Abstract

*Background and Objectives*: This study analyzed the frequency of factors influencing the course and outcomes of out-of-hospital cardiac arrest (OHCA) in Serbia and the prediction of pre-hospital outcomes and survival. *Materials and Methods*: Data were collected during the period from 1 October 2014, to 31 September 2023, according to the protocol of the EuReCa_One study (clinical trial ID number NCT02236819). *Results*: Overall 9303 OHCA events were registered with a median age of 71 (IQR 61–81) years and 59.7% of them being males. The annual OHCA incidence was 85.60 ± 20.73/100,000. Within all bystander-witnessed cases, bystander-initiated cardiopulmonary resuscitation in 15.3%. Within the resuscitation-initiated group, return-of-spontaneous circulation (ROSC) on scene (any ROSC) was present in 1037/4053 cases (25.6%) and ROSC on admission to the nearest hospital in 792/4053 cases (19.5%), while 201/4053 patients survived to hospital discharge (5.0%). Predictive potential on pre-hospital outcomes was shown by several factors. Also, of all patients having any ROSC, 89.2% were admitted to the hospital alive. The probability of any ROSC dropped below 50% after 17 min passed after the emergency call and 10 min after the EMS scene arrival. These time intervals were significantly associated with survival to hospital discharge (*p* < 0.001). Five-minute time intervals between both emergency calls and any ROSC and EMS scene arrival and any ROSC also had a significant predictive potential for survival to hospital discharge (*p* < 0.001, HR 1.573, 95% CI 1.303–1.899 and *p* = 0.017, HR 1.184, 95% CI 1.030–1.361, respectively). *Conclusions*: A 10-min time on scene to any ROSC is a crucial time-related factor for achieving any ROSC, and indirectly admission ROSC and survival to hospital discharge, and represents a golden time interval spent on scene in the management of OHCA patients. A similar effect has a time interval of 17 min from an emergency call. Further investigations should be focused on factors influencing these time intervals, especially time spent on scene.

## 1. Introduction

Out-of-hospital cardiac arrest (OHCA) is a serious public health issue globally. More than 356,000 cardiac arrest cases are annually registered in the United States [1] and approximately 275,000 in Europe [2]. Despite significant efforts undertaken by numerous countries and scientific groups, OHCA survival in Europe has not shown significant changes in recent decades. Therefore, various studies, including the European Registry of Cardiac Arrest (EuReCa), try to find solutions to increasing survival rates in these patients. Early reaction is one of the key factors influencing outcomes in these patients [3]. According to the latest European Resuscitation Council’s (ERC) guidelines, the chain of survival includes broader general population enrollment to the early recognition of cardiac arrest, early cardiopulmonary resuscitation (CPR) initiation, and the early application of direct-current (DC) shock [4,5]. The “EuReCa_One” was the first study that collected data into a single database from 27 countries across Europe [6,7]. As such, OHCA has been monitored in the Republic of Serbia since 2014 when numerous Serbian healthcare institutions joined the EuReCa_ONE project [8]. With the presence of the collected data, it became possible to compare the obtained results for a period of one decade with other European and non-European regions.

The EuReCa registry aims to observe the process of patient management, including the outcomes of OHCA in a large number of European countries. In this prospective analysis, special attention is drawn to the return of spontaneous circulation (ROSC), hospital admission with ROSC, and 30-day survival [7,8,9,10,11].

Although OHCA represents a challenge, public health authorities in Serbia did not face it until 2014. With the enrollment of the Serbian Resuscitation Council (Novi Sad, Serbia) in the EuReCa_One study, the comprehensive collection of data and epidemiological follow-up of OHCA according to the Utstein protocol has been initiated [12]. Initial data evaluation showed that there is a necessity for prolonged data collection in order to improve the access and care of OHCA patients. Fortunately, the continuation of data collection of OHCA in Serbia was supported by the Serbian Resuscitation Council after authorization was given by ERC.

During the last decade, EuReCa data became the basis of observation of epidemiological trends and effects of activities undertaken towards quality improvement of management of OHCA patients [8,12,13,14,15,16,17,18,19,20,21].

The aim of this study is to determine factors influencing survival on hospital admission, as well as to analyze the influence of pre-hospital factors on survival to hospital discharge in patients with OHCA in Serbia.

## 2. Materials and Methods

Data on OHCA for this prospective observational multicentric study were collected during the period from 1 October 2014, to 31 September 2023, according to the protocol of the EuReCa_One study registered at the United States National Library of Medicine’s (Bethesda, MD, USA) registry of clinical trials under the ID number NCT02236819 [6,22]. According to the EuReCa_One study protocol, prior to the inclusion of the Serbian registry into the EuReCa_One study, written approval from each participating emergency medical service (EMS) center was obtained, clearly describing the permission to use and transmit defined data for research purposes on an international basis. EMS centers in Serbia were enrolled on a voluntary basis which was followed by entering the data into the unique electronic database by the main investigator of each enrolled EMS center. The study was conducted in accordance with the Declaration of Helsinki and approved by the Ethics Committee of Serbian Resuscitation Council (approval number A—034-150614-2014) on 15 June 2014 according to the EuReCa_One study protocol, which also waived participants’ informed consent collection.

The study included data on all-cause OHCA in both adult and pediatric patients confirmed by EMS where EMS intervention was present within the geographic and administrative areas covered by the Serbian EMS centers enrolled in the study. The data were collected in a comprehensive database and analyzed according to the Utstein protocol [23,24,25].

Data confidentiality and coding were applied according to the EuReCa_One study protocol describing all segments of patient demographic and management-related data observed [6,22].

All data determined by EuReCa protocol and collected by the investigation centers in Serbia and validated by the main investigators have been included in the analysis. Descriptive statistical models were used to evaluate the incidence of OHCA as well as to analyze the performance of CPR measures applied by witnesses, main prehospital outcomes, and survival to hospital discharge. The distribution of numerical variables data was examined by the Kolmogorov–Smirnov test with Lilliefors significance correction. Median values with interquartile range (IQR) were utilized as measures of central tendency and representative values for all numerical variables, based on the normality of data distribution, while frequency and percentage were used to describe categorical data. Incidence was calculated using the population covered and extrapolated to incidence rates per 100,000 population per year. The Mann–Whitney U test was used to compare means and mean ranks of numerical variables, while the Chi-square test and Fisher’s exact test were used to analyze the association between categorical variables. Univariable and multivariable binary logistic regression analysis, log-rank test, and the Cox proportional hazards model were utilized to define independent predictors of outcome events before admission to the hospital (shockable initial heart rhythm, any ROSC, and ROSC on hospital admission), as well as survival at hospital discharge. The analysis was performed by using Statistical Product and Service Solutions package for Windows v26.0 (IBM, Armonk, NY, USA).

## 3. Results

The EuReCa_Serbia 2014–2023 encompassed EMS centers from 16 municipalities in Serbia with a total covered population of 1.2 million in 2014, which represents 18.55% of the population of Serbia (the population size was determined based on the last census conducted prior to study initiation).

**Figure 1:**
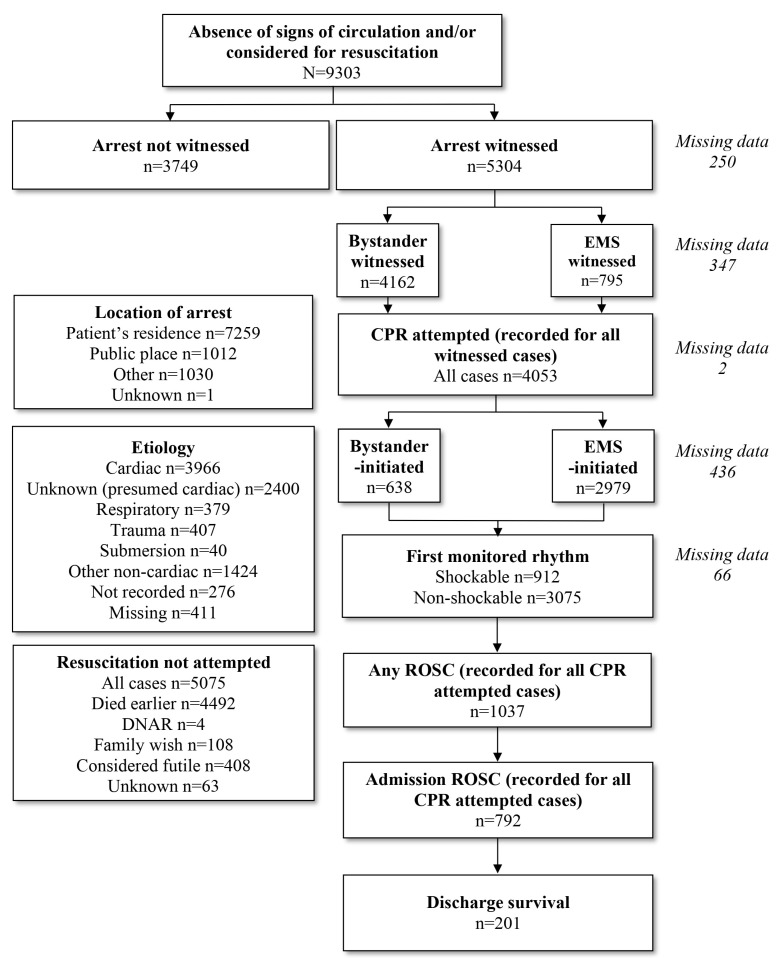
Flowchart of OHCA-related data analysis (Legend: EMS—emergency medical service; DNAR—do not attempt resuscitation; CPR—cardiopulmonary resuscitation; ROSC—return of spontaneous circulation).

Within the study period, we observed 9303 OHCA events defined by the EuReCa_One study protocol, with an average annual OHCA incidence of 85.60 ± 20.73/100,000. Of all registered OHCA patients, 5557/9303 were males (59.7%; 107.91 ± 27.86/100,000/annum) and 3746/9303 were females (40.3%; 64.27 ± 19.11/100,000/annum). The median age of all patients was 71 years (IQR 61–81). The median age of female patients with OHCA was significantly higher compared to male patients (Med 71 IQR 62–79 vs. Med 66 IQR 57–74, *p* < 0.001). The flow diagram of patients included in the study is presented in Figure 1, while data on the cause and location of OHCA events are presented in Table 1.

Out of all the registered OHCA events, more than half (*n* = 5304/9303; 57.0%; 53.87 ± 21.31/100,000/annum) were witnessed and CPR was attempted in 4053/9303 cases (43.6%; 44.77 ± 22.34/100,000/annum). Within that group of patients, CPR was initiated by bystanders in 638/4053 cases (15.1%; 6.60 ± 3.98/100,000/annum). Within the group of initiated CPR, cardiac etiology was observed in 3386/4053 cases (80.2%) and initial heart rhythm was shockable in 912/4053 patients (22.5%). ROSC on scene (any ROSC) was present in 1037/4053 cases (25.6%) and ROSC on admission to the nearest hospital in 792/4053 cases (19.5%). In the same group, 201/4053 patients survived hospital discharge (4.96%; 1.81 ± 2.39/100,000/annum). The annual incidence of outcome events per 100,000 population is presented in Table 2.

Variables associated with shockable initial heart rhythm, any ROSC, hospital admission ROSC, and survival to hospital discharge were:

- population size greater than 100,000 inhabitants (vs. less than 100,000),
- patient age of 65 years or less (vs. more than 65 years),
- female sex (vs. male),
- cardiac OHCA etiology (vs. non-cardiac),
- out-of-residence OHCA location (vs. residence),
- EMS-witnessed OHCA (vs. bystander-witnessed),
- EMS-initiated CPR (vs. bystander-initiated),
- dispatcher-assisted bystander-CPR (vs. dispatcher-non-assisted), and
- full bystander-CPR (vs. cardiac compressions only—CCO).

The distribution of prehospital outcome variables (shockable initial rhythm, any ROSC, and admission ROSC) within groups of prehospital predicting factors, as well as their association with the occurrence of shockable initial heart rhythm, any ROSC and ROSC on hospital admission are presented in Table 3.

When OHCA occurred in a patient’s residence collapse was witnessed in 57.0% of cases, which is significantly less frequent compared to the 64.4% witnessing rate in out-of-residence OHCA cases (*p* < 0.001).

In the group of witnessed OHCA events, CPR was initiated more often in patients 65 years of age or less, compared to those older than 65 years (83.1% vs. 72.0%, respectively; *p* < 0.001). In the same group of patients, CPR was initiated more often in males compared to females (80.1% vs. 70.5%, respectively; *p* < 0.001). In witnessed OHCA cases occurring in out-of-residence locations, CPR was initiated in 89.3% of cases, and in 72.4% of cases occurring in the patient’s residence (*p* < 0.001). These three factors (patient age of 65 years or less, female sex, and out-of-residence OHCA location) have been observed in this study as independent predictors of initiating CPR in witnessed cases, which was confirmed with multivariable binary logistic regression analysis (*p* < 0.001, 95% CI 1.497–1.785, OR 1.635 for patient age of 65 years or less; *p* < 0.001, 95% CI 0.692–0.823, OR 0.755 for female sex and *p* < 0.001, 95% CI 1.665–2.040, OR 1.843 for out-of-residence OHCA location). 

In the group of CPR-attempted OHCA cases, variables showing significant association with the investigated pre-hospital outcomes were included in the univariable binary logistic regression model. Those showing significant predictive potential were further included in the multivariable regression model, providing the list of independent predictors of investigated pre-hospital outcome events (Table 4).

Significant independent predictors of shockable initial heart rhythm were a municipality population smaller than 100,000 inhabitants, male sex, cardiac OHCA etiology, and out-of-residence OHCA location. In the group where bystander-initiated CPR performed full CPR measures applying CCO was also a positive predictor of shockable initial rhythm (Table 4).

Independent predictors of any ROSC were patient age less or equal to 65 years, out-of-residence OHCA location, non-cardiac cause of OHCA, and within the bystander-initiated CPR group, full CPR methods applied vs. CCO. Dispatcher assistance in the group of patients when bystander-initiated CPR was observed as a negative predictor of any ROSC (Table 4).

ROSC present at hospital admission was independently predicted by out-of-residence OHCA location and size of the municipality where OHCA events occurred smaller than 100,000 inhabitants. The cardiac cause of OHCA was observed as a negative independent predictor of admission ROSC (Table 4).

In the group of CPR-attempted OHCA cases, shockable initial heart rhythm was also a significant predicting factor for both any ROSC (OR 5.461) and admission ROSC (OR 4.434).

Pre-hospital outcomes were also compared between the group of all witnessed cases and the Utstein comparator group. The criteria for patient inclusion into the Utstein comparator group were adult patients (18 years of age or older) with cardiogenic or presumed cardiogenic OHCA where CPR was attempted, and shockable rhythm was the first observed heart rhythm. In the group of all witnessed OHCA cases, shockable initial heart rhythm occurred in 21.4% of patients, any ROSC in 24.6%, and admission ROSC in 18.8%. In the Utstein comparator group, any ROSC was achieved in 52.1% of cases and admission ROSC in 41.3% of cases (2.1- and 2.2-fold increased prevalence compared to the group of all witnessed cases, respectively).

### Influence of Time-Related Factors and Distance to Nearest Hospital on Pre-Hospital Outcomes and Survival to Hospital Discharge

We analyzed the influence of time intervals between emergency calls and any ROSC, as well as EMS scene arrival and any ROSC on both prehospital survival rates and hospital discharge survival rates. Out of all patients with any ROSC observed, 89.2% were admitted to the nearest hospital alive. This implies that any ROSC is a crucial factor for hospital admission and, therefore, hospital discharge, and was defined as the main pre-hospital survival outcome in this analysis. Hospital discharge survival was the main long-term survival outcome event.

**Figure 2:**
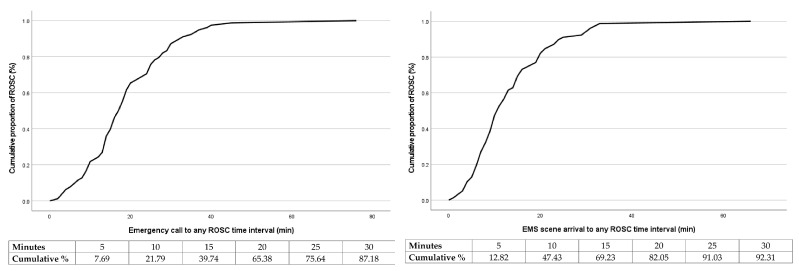
Cumulative proportion of return of spontaneous circulation (%) over time (min) in patients with out-of-hospital cardiac arrest—Return of spontaneous circulation was achieved in 50% of patients within 17 min after emergency call (**left**) and 10 min after emergency medical team arrival on scene (**right**) (Legend: ROSC—return of spontaneous circulation).

The probabilities of achieving any ROSC relative to the time between the emergency call and any ROSC, as well as EMS scene arrival and any ROSC are presented in Figure 2. The probability of any ROSC drops below 50% after 17 min passed after the emergency call and 10 min after the EMS scene arrival.

A 17-min time point of the emergency call to any ROSC time interval was significantly associated with survival to hospital discharge. In cases when this time interval was 17 min or less, 56.0% of patients survived to hospital discharge, while 28.0% in the group when this time interval was longer than 17 min (*p* < 0.001). The same finding was observed when comparing a 10-min time point of EMS scene arrival to any ROSC time interval (56.0% vs. 28.0%, *p* < 0.001).

**Figure 3:**
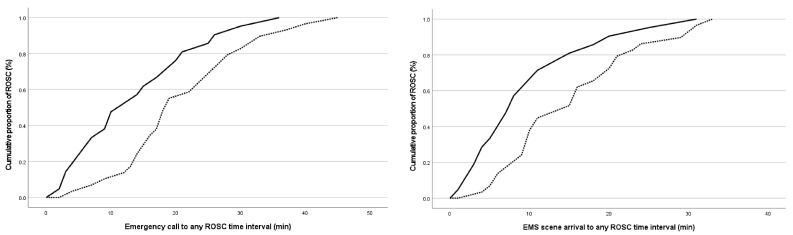
Cumulative proportion of return of spontaneous circulation (%) over time passed from emergency call (**left**) and emergency medical team arrival on scene (**right**) (min) in out-of-hospital cardiac arrest patients with survival to hospital discharge and patients who died, compared with log-rank test (*p* < 0.001 and *p* < 0.001, respectively) (Legend: ROSC—return of spontaneous circulation; solid line—survived to discharge; dotted line—died).

Patients who survived hospital discharge had significantly shorter emergency calls to any ROSC, as well as EMS scene arrival to any ROSC time interval (*p* < 0.001) (Figure 3).

**Figure 4:**
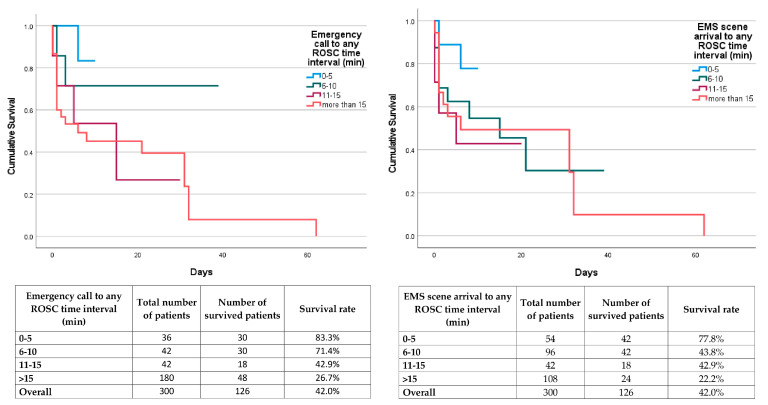
Hospitalization duration and rate of survival to hospital discharge in out-of-hospital cardiac arrest patients related to emergency call and emergency medical service arrival on scene to any ROSC time intervals (Legend: ROSC—return of spontaneous circulation; EMS—emergency medical service).

The log-rank test showed a statistically significant difference in in-hospital survival time between different groups representing 5-min time intervals between both emergency call and any ROSC and EMS scene arrival and any ROSC (*p* < 0.001 and *p* < 0.001, respectively). Cox regression proportional hazard model also showed that 5-min time intervals between both emergency call and any ROSC and EMS scene arrival and any ROSC are significant predictors of survival to hospital discharge (*p* < 0.001, HR 1.573, 95% CI 1.303–1.899 and *p* = 0.017, HR 1.184, 95% CI 1.030–1.361, respectively) (Figure 4).

The median number of hospitalization days in all patients, patients who survived to hospital discharge, and patients who died during hospitalization, as well as the maximum duration of hospitalization in different 5-min time intervals between both emergency call and any ROSC and EMS scene arrival and any ROSC are presented in Table 5.

Survival to hospital discharge was more frequently present in the Utstein group of patients, compared to the non-Utstein group (75.2% vs. 33.4%, respectively, *p* < 0.001). Fulfilling the criteria for an Utstein event was observed as a significant predictor for hospital discharge survival (*p* = 0.018; OR 1.957; 95% CI 1.123–3.412).

In the group of OHCA events where the distance to the nearest hospital was less than 5 km, 26.7% of patients survived to hospital discharge, while only 7.0% survived in the group where that distance was 5 km or more (*p* = 0.004). Binary logistic regression showed a predictive potential of distance to the nearest hospital of 5 km or more on survival to hospital discharge (*p* = 0.011, OR 0.206, CI 0.061–0.694).

## 4. Discussion

The current observational study analyzed the data collected during the period from 1 October 2014 to 31 September 2023 according to the Utstein protocol and EuReCa project methodology [9,10,23,24,25] which makes it comparable to the reports of the studies following the same methodology. The results show that the annual OHCA incidence rate in Serbia is 85.60 ± 20.73/100,000. Previous reports from the EuReCa_Serbia registry of OHCA incidence vary throughout the years of data collection (49.5–232.1/100,000/annum) [15,16,17,20] but are comparable to the reports on data in different European countries published in the epidemiology report of the ERC 2021 Guidelines (range 67–170/100,000/annum) [5].

Serbian reports are also comparable with national reports from various countries. Previously published results from individual European countries showed variations in OHCA incidence among different countries, ranging from 18.6 to 34.0/100,000/annum in Spain [26,27], 57.0 to 59.0/100,000/annum [28] to 230.0/100,000/annum in the Czech Republic [29]. Reports from other countries also show variations between different time periods and between different reports for the same country. A Danish report observed annual incidence for five different regions in Denmark ranging from 32.9 to 42.4 per 100,000 inhabitants [30]. Another 2022 report from the Danish OHCA Registry showed an annual incidence of 93.0 per 100,000 inhabitants [31]. Another example is recent Polish reports [32,33,34], where the same observations showed different values in different periods of time. The 2016 study from Poland which analyzed the data from 2013 reported an OHCA frequency of 170.0/100,000/annum [32]. A slightly lower result from the same country was observed during the period 2006–2007 (156.0/100,000/annum), although that analysis was performed on OHCA patients with presumed cardiac etiology only [33]. However, the report from the same country analyzing data during 2018 presented an annual incidence range of 58.9–84.5/100,000 among different provinces [34]. Our results are comparable with non-European reports as well. For example, a 2023 study from China reports an annual OHCA incidence of 95.7/100,000 inhabitants [35]. The mentioned time-related and geographical differences among different countries and within individual countries can be explained by different approaches to data collection methodology, as well as the organization of the prehospital emergency system and its function. The same reason could be the explaining factor for the time difference in OHCA incidence rate in some other European countries when analyzing data from different periods of time.

The results showed a five-year difference in median age between male and female patients (66 vs. 71 years) in favor of females. Previous results rarely reported the age differences of OHCA patients related to their sex, but showed similar findings, although with a smaller age difference. A North American research group showed a 2.2-year difference in 2021 [36], while that difference was 2.9 years (67.4 vs. 64.5 years) in a recent Canadian report [37]. EuReCa_Two study analysis reported a mean age of male OHCA patients of 66 years [10], the same as the median age of male patients in this current report. Some studies, however, analyzed between-sex age differences relative to the outcomes. One of them is a study published in Lancet in 2022, reporting the median age of male OHCA patients who died at the scene and were transported to the hospital in the male subgroup of 67 and 64 years, respectively, and the median age of female OHCA patients of 77 and 73 years, respectively [35]. Observing the range of these results, the age reported in that study is even larger compared to the current report.

Related to the location of the OHCA event, our results show that most of the OHCA events are observed in a patient’s residence (7259/9303 of cases; 78.0%; 66.73 ± 13.12/100,000/annum) which is comparable to the average percentages of previously published reports (69.4% in EuReCa_One study, 70.2% in EuReCa_Two study, 75% in 2019 French report, 66%, 64%, and 64% for three consecutive 5-year intervals in 15-year German report published in 2023, 82.6% in 2024 Saudi Out of Hospital cardiac Arrest Registry results, and 79.2% in Chinese 2022 report) [9,10,35,38,39,40].

EuReCa_Serbia reports an OHCA witnessing rate of 5304/9303 (57.01%) OHCA cases (53.87 ± 21.31/100,000/annum) with annual variations [15,16]. The current finding is lower than the EuReCa_Two Report (66.6%) [10], the percentage reported in the 2016 studies from Poland (60%) [32], but higher than the report from the United Kingdom (53%) [41] and the United States where 40.9% of adult OHCA events were witnessed [1].

Bystander-witnessing OHCA was reported in 4162 of cases (41.49 ± 17.63/100,000/annum), which represents 44.74% of all 9303 registered OHCA events included in this study, which is lower compared to the 13-year Swiss study published in 2016 (69.0%) [42] and the result shown in the Irish National Registry 2019 Annual Report (50.0%) [43].

In this study, in the group of all witnessed OHCA cases, CPR was attempted in 4053/5304 (76.4%; 44.77 ± 22.34/100,000/annum) patients. In that group of CPR-attempted OHCA cases, the first observed heart rhythm was shockable (ventricular fibrillation/pulseless ventricular tachycardia) in 912/4053 (22.5%). Similar results were also observed by the EuReCa_Two study, reporting the overall average value of detection of shockable initial heart rhythm in CPR-attempted OHCA cases was 20.2%, the median of the country value of 19.2%, and a range of 11.4–36.8% of cases [10].

Although there is a high percentage of witnessing OHCA, in the group of 4162 bystander witnessed all-cause OHCA cases analyzed in this study, there is still a small number of bystander-initiated CPR cases. CPR was attempted by bystanders in 638 cases (15.3%; 6.60 ± 3.98/100,000), which is lower than some other European national reports, like the 2017 report from the Spanish OHCA registry (24.2%) [44]. Differences in study methodology and data collection could also be the explanation for these variations in bystander-CPR-related results.

In this report, we observed 1037/4053 (24.6%) cases with any ROSC, with an average annual incidence rate of 10.81 ± 7.73/100,000. The percentage value of the same finding in this study (24.6%) is higher than the result of some of the published reports, such as the Irish National Registry 2019 Annual Report [43]. The result observed in this study is within the range of the country values reported in both EuReCa_One and EuReCa_Two study findings (9.1–50.0 and 6.9–43.3, respectively), but still lower than the median of the country values that the studies reported (30.6% and 29.7%, respectively) [9,10].

In the current study, any ROSC was observed in 1037 of 4053 CPR-attempted OHCA cases (25.6%; 10.81 ± 7.73/100,000/annum). In the group of initially detected shockable heart rhythm, it was observed in 476/912 (52.2%), while in the non-shockable rhythm group, it was observed in 517/3100 (16.7%) of cases which emphasizes the association of shockable rhythm and any ROSC.

In all the CPR-attempted OHCA events, significant independent predictors of shockable initial heart rhythm were municipality population size, patient sex, cause and location of OHCA, as well as the type of CPR provided by bystanders. The probability of shockable initial heart rhythm increased 1.84 times in cases where OHCA occurred in municipalities with less than 100,000 inhabitants, 1.76 times in male patients, 3.77 times in cardiac cause OHCA events, 2.89 times in out-of-residence OHCA events, and 2.27 times in bystander-CPR-initiated OHCA events when a bystander performed full CPR (compared to cardiac compressions only). Many previous studies investigated the predictive potential of various factors on shockable initial heart rhythm in OHCA patients. A 2017 Taiwanese study highlighted witnessed status, male sex, age less than 65 years, and public location of OHCA events as independent predictors of OHCA [45]. Observations in this study, however, showed a higher predictive potential of the male sex on shockable rhythm probability (2.45 times more frequent occurrence vs. 1.76 times in the present study). Also, we did not find the predictive potential of patient age on shockable rhythm. Male sex and out-of-residence events were also shown as predictors of this outcome in another study conducted in Denmark in 2016 [46].

In all CPR-initiated OHCA events, significant independent predictors of any ROSC were patient age, cause and location of OHCA, dispatcher assistance, type of CPR provided by the bystander, as well as the shockability of the initial heart rhythm. The probability of any ROSC had a 1.80-fold increase in patients 65 years of age or younger, a 2.94-fold increase in non-cardiac cause OHCA events, 2.55-fold in out-of-residence OHCA events, 1.54-fold in non-DA-assisted bystander-CPR-initiated OHCA events, 1.61-fold in bystander-CPR-initiated OHCA events when the bystander performed full CPR (compared to cardiac compressions only), and 5.46-fold in cases with shockable initial heart rhythm. We did not observe the predictive potential of patient sex on any ROSC, although some previous studies showed that this outcome is predicted by female sex [47].

In the group of CPR-attempted OHCA cases, shockable initial heart rhythm independently increases the chance for any ROSC 5.5 times and admission ROSC 4.4 times, and that indirectly influences the chances for survival. A recently published Iranian study reported a 1.86-fold increase in ROSC when a shockable rhythm is present [48]. Also, a recent Italian report shows an 8.18-fold increase in admission ROSC chance when the shockable rhythm is present [49].

Of 4053 CPR-attempted all-cause OHCA cases, admission ROSC was observed in 792 cases (19.5%; 8.18 ± 5.05/100,000/annum) which is similar to both EuReCa_One and EuReCa_Two studies reporting this frequency of 24.3% and 25.3%, respectively [9,10]. In the present study, only patients with any ROSC were transported to the hospital. Therefore, all of the patients admitted to a hospital with ROSC had any ROSC. Of all patients with any ROSC, 89.2% had sustained ROSC on hospital admission.

In our study, in all CPR-initiated OHCA events, significant independent predictors of admitting OHCA patients alive to the hospital were municipality population size, cause, and location of OHCA, as well as the shockability of the initial heart rhythm. The probability of admission ROSC showed a 2.14-fold increase in municipalities with less than 100,000 inhabitants, a 2.06-fold increase in non-cardiac cause OHCA events, a 1.59-fold increase in out-of-residence OHCA events, and a 4.43-fold increase in cases with shockable initial heart rhythm.

Univariable binary logistic regression model analysis with multivariable regression showed that patients having OHCA in municipalities with 100,000 inhabitants or less have still higher chances for achieving shockable initial heart rhythm (1.8-fold) and to be transported to a hospital with ROSC (2.1-fold). The mentioned results emphasize that life in smaller municipalities has a benefit followed by two-fold greater survival chances on hospital arrival. We did not observe an influence of population size on survival on hospital discharge. Similarly, one of the recent studies showed no association between air pollution in highly populated areas and long-term survival rate [50].

Compared to men, women have a 1.76-fold decreased probability of having a shockable initial rhythm, while patients 65 years of age or younger have almost doubled chances (1.80-fold increase) of achieving any ROSC, compared to those older than 65 years of age.

Interestingly, patients with cardiogenic OHCA have an almost 3.8-fold increased chance of achieving shockable initial rhythm, but the chances for any ROSC and keeping ROSC until hospital admission significantly drops in these patients, being more than twice as low compared to those with non-cardiac cause OHCA.

Out-of-residence location of OHCA significantly increases the chances of attaining all three investigated positive pre-hospital outcome events (shockable initial rhythm 2.9-fold, any ROSC 2.6-fold, and admission ROSC 1.6-fold), as it is an expected finding due to the higher exposure level to a prompt reaction by bystanders in providing basic life support measures and making an emergency call.

In the CPR-attempted group of OHCA patients, the fact whether the bystander initiated CPR or not did not have a significant influence on achieving shockable initial rhythm and any ROSC. In cases when bystander-initiated CPR, admission ROSC was achieved in 27.1% of all CPR-attempted cases, while the same outcome was achieved in 20.9% of cases when EMS commenced CPR measures. ERC guidelines emphasize the inclusion of bystanders in providing CPR measures, highlighting the fact that any CPR measures are better than not providing measures at all [5]. These EuReCa_Serbia results for the period 2014–2023 partially support these observations related to hospital arrival.

When analyzing the completeness of the applied CPR measures by bystanders in the group of bystander-initiated CPR performing full CPR measures compared to CCO, the chances for achieving shockable initial heart rhythm were 2.3 times and any ROSC 1.6 times. Differences are also observed in the EuReCa_Two study which reports 35% of any ROSC when bystanders were performing full CPR measures and 26% when performing CCO [51].

Also, our study showed that patients with Utstein events have a 2.1- and 2.2-times greater chance of any ROSC and admission ROSC, respectively, compared to all witnessed cases. That event is also significantly associated with hospital discharge survival, giving a two-fold increase in the probability of that outcome.

The rate of survival on hospital discharge the present study reports (4.96%) is smaller than the one reported in the EuReCa_Two study (8%), but within the range of the same report (0–18%) [10].

In our study, the probability of any ROSC dropped below 50% after 17 min passed after the emergency call and 10 min after EMS arrival at the scene. These two time points were significantly associated with survival to hospital discharge. Also, in cases when the emergency call to any ROSC time interval was 10 min or shorter, 76.9% of survival-to-discharge rate was observed, compared to 29.7% when that interval was longer than 10 min. Similarly, in cases when EMS arrival at the scene to any ROSC time interval was 10 min or shorter, 56.0% of survival-to-discharge rate was observed, compared to 28.0% when the same interval was longer than 10 min. Five-minute time intervals between both emergency calls and any ROSC and EMS scene arrival and any ROSC had a significant predictive potential for in-hospital survival. With each 5-min time interval between an emergency call and any ROSC prolongation, a 1.57-fold increase in in-hospital mortality rate was observed, while a 1.18-fold increase was noted with each 5-min time interval between EMS arrival at the scene and any ROSC prolongation. Additionally, the maximum number of hospitalization days was the lowest in the groups of patients when both of these two time intervals lasted up to five minutes.

A few studies investigated the influence of time-to-ROSC and survival of OHCA patients. Grunau et al. reported that 50% of OHCA survivors achieve any ROSC within eight minutes from the commencement of CPR and recommended transporting of patients between 8 and 24 min of professional on-scene CPR [52]. Also, three reports from the Netherlands showed significantly lower time-to-ROSC in 30-day survivors compared to non-survivors and reported the highest survival rate in cases transported within 20 min of on-scene time, and suggested initiating patient transport between 8 and 15 min after EMS arrival at the scene [53,54,55]. A similar influence on any ROSC occurrence was also observed in pediatric patients with OHCA [56].

A Korean study published in 2021 showed that in patients with refractory OHCA having a shockable initial heart rhythm, continuing CPR for more than 15 min on scene is associated with a decreased chance of survival and good neurological outcome [57].

The distance to the nearest hospital was also observed as a significant predictor of survival to hospital discharge. When the distance was 5 km or longer, the probability of surviving until hospital discharge decreased by 79.4% which corresponds to our previous findings [21].

We did not find a predictive potential of pre-hospital factors on survival to discharge which emphasizes the role of previously extensively discussed hospitalization-related factors in predicting outcomes in OHCA patients after hospital admission [58].

The main shortcomings of this study include its limitation to only those parameters defined by the EuReCa protocol as it does not include some which have been previously shown to have an importance in influencing predominantly pre-hospital, but also long-term outcomes, as well as some outcome-related parameters (adrenalin application time and dose, number of DC shocks applied, neurological outcome and scores used to evaluate the neurological outcome, such as cerebral performance category, etc.) [59,60,61]. Also, this study could not assess the quality of applied CPR measures by both bystanders and EMS (frequency and depth of cardiac compressions, quality of ventilations, etc.) which could also have an influence on both pre-hospital and long-term outcomes due to the absence of data related to those parameters in the main questionnaire defined by EuReCa protocol. Finally, parameters related to the in-hospital management of these patients are also not covered by the same protocol and were not analyzed in this study as parameters with an influence on long-term outcomes. Therefore, the possibility that those parameters could have an impact on the influence of pre-hospital factors on the same outcome exists. This is especially true since it has been proven previously that in-hospital factors (such as target temperature management, oxygen therapy, and ventilatory settings) are important in predicting long-term survival and neurological outcomes in these patients [58]. Although this study indirectly showed that factors other than pre-hospital take charge in determining the probability for long-term outcomes, further analyses encompassing these, as well as other mentioned limitations, should fill the gaps in understanding the influence of all the other potential variables not covered by this study.

Additionally, there is scarcity in certain areas of data collection. The voluntary nature of EMS center inclusion carries a risk of the lack of homogeneity of data since EMS centers that entered this study were most probably the ones being ready to be enrolled and to present their data. Therefore, considering regional variabilities, there is a great probability that this study represents mainly those areas where improvements are being made daily and where professional knowledge and skills of personnel are at a high level with a constant potential for improvement. Besides, due to a frequent fluctuation in the EMS team personnel members who performed data collection in this study, a constant renewal of training was needed which could have influenced the data collection consistency not having been at a satisfactory level in all segments of the study. This could be further influenced by a long follow-up time period. Time-related factors could also be influenced by several factors not being analyzed in this study, such as equipment used, organization of EMS teams, and training of their members. It is difficult to assess the process of applying basic life support measures by EMS teams since there are no data describing which guidelines EMS teams apply.

Also, in this study, we analyzed survival to hospital discharge as the main survival outcome. However, different studies investigated similar predictors on different outcomes, the majority of them showing similar influence on both survival to discharge and 30-day survival as well as equivalence between survival to hospital discharge and 30-day survival as a primary long-term survival outcome in OHCA patients [62]. This should also be further confirmed by studies in the future.

## 5. Conclusions

The current understandings indicate an association between the duration of cardiac arrest and survival.

This study emphasizes the significance of any ROSC and the crucial impact of a time interval of 17 min from an emergency call to any ROSC and 10 min from EMS arrival at the scene. These time intervals significantly influence any ROSC occurrence and strongly predict the duration of hospitalization and survival to hospital discharge.

This information should encourage all EMS personnel members to apply CPR measures decisively and persistently, as suggested by findings of the International Liaison Committee on Resuscitation (ILCOR) scientific consensus, and not to terminate their application until the last possibility for the occurrence of ROSC is exhausted.

This study scientifically proves the justification of this statement and the application of our findings (persistent application of CPR measures) in practice will hopefully increase the frequency of outcomes related to survival on hospital discharge and thereby save more lives.

There is also a need to design further investigations which will result in detailed findings, and thus improve the understanding of factors influencing these time intervals, especially time spent on the scene (equipment, team characteristics, training, etc.).

## Figures and Tables

**Table 1:** Distribution of OHCA cause and location categories in emergency medical service-confirmed out-of-hospital cardiac arrest in Serbia.

Cause	N (%)
Not recorded	276 (3.0)
Cardiac	3962 (42.6)
Unknown (presumed cardiac)	2400 (25.8)
Total cardiac	6362 (68.4)
Trauma	407 (4.4)
Submersion	40 (0.4)
Respiratory	379 (4.1)
Other non-cardiac	1424 (15.3)
Missing data	411 (4.4)
**Location**	**N (%)**
Residence	7259 (78.0)
Long term care	504 (5.4)
Workplace/office	90 (1.0)
Street	727 (7.8)
Public building	268 (2.9)
Sports facility	17 (0.2)
Outpatient hospital	23 (0.3)
Ambulance car	11 (0.1)
Other	402 (4.3)
Unknown	1 (0.0)

**Table 2:** Frequency and annual incidence rates of out-of-hospital cardiac arrest-related events.

Variable	N	Per 100,000/Annum (Mean ± SD)
OHCA	9303	85.60 ± 20.73
Residence as OHCA location	7259	66.73 ± 13.12
Public place	1012	10.19 ± 7.89
Other location	628	5.00 ± 6.66
All witnessed	5304	53.87 ± 21.31
Witnessed by bystander	4162	41.49 ± 17.63
Witnessed by EMS	795	8.12 ± 4.59
CPR Attempted	4053	44.77 ± 22.34
Bystander-initiated CPR	638	6.60 ± 3.98
EMS-initiated CPR	2979	26.77 ± 10.50
Any ROSC	1037	10.81 ± 7.73
Admission ROSC	792	8.18 ± 5.05
Hospital discharge survival	201	1.81 ± 2.39

Legend: SD—standard deviation; OHCA—out-of-hospital cardiac arrest; EMS—emergency medical service; CPR—cardiopulmonary resuscitation; ROSC—return of spontaneous circulation.

**Table 3:** Association between investigated factors and pre-hospital outcomes of out-of-hospital cardiac arrest.

Variables		Shockable Rhythm N (%)	*p* Value	Any ROSC N (%)	*p* Value	Hospital Admission ROSC N (%)	*p* Value
Population	≤100,000	377 (31.3)	<0.001	378 (30.5)	<0.001	291 (58.7)	<0.001
	>100,000	525 (18.9)		656 (22.2)		499 (28.9)	
Patient age	≤65 years	521 (29.9)	<0.001	502 (27.4)	<0.001	377 (36.9)	0.242
	>65 years	383 (17.1)		535 (22.7)		415 (34.5)	
Patient sex	Male	649 (25.1)	<0.001	672 (24.6)	0.807	519 (35.5)	0.983
	Female	255 (18.4)		365 (24.9)		273 (35.6)	
Etiology of OHCA	Cardiac	881 (26.3)	<0.001	792 (23.6)	<0.001	628 (33.4)	<0.001
	Non-cardiac	17 (2.9)		199 (33.7)		158 (48.9)	
OHCA location	Residence	547 (19.1)	<0.001	560 (18.6)	<0.001	415 (27.4)	<0.001
	Out of residence	357 (32.1)		477 (40.4)		377 (52.7)	
Bystander-witnessed or EMS-witnessed	Bystander	597 (21.6)	<0.001	637 (21.8)	<0.001	472 (31.0)	<0.001
	EMS	248 (34.5)		338 (44.9)		275 (56.4)	
Bystander CPR (within bystander witnessed group)	Yes	216 (35.4)	<0.001	224 (35.1)	<0.001	172 (53.9)	<0.001
	No	662 (20.6)		796 (23.4)		606 (33.8)	
Dispatcher-assisted CPR (within bystander CPR group)	Yes	89 (32.5)	<0.001	96 (32.9)	0.003	88 (50.6)	<0.001
	No	796 (22.7)		921 (25.0)		687 (36.7)	
CCO or full CPR (within bystander CPR group)	CCO	69 (28.9)	<0.001	80 (32.4)	0.019	68 (50.7)	0.741
	Full	97 (45.5)		98 (42.8)		66 (52.8)	

Legend: ROSC—return of spontaneous circulation; OHCA—out-of-hospital cardiac arrest; EMS—emergency medical service; CPR—cardiopulmonary resuscitation; CCO—cardiac compressions only.

**Table 4:** Independent predictors of prehospital outcomes of out-of-hospital cardiac arrest (multivariable regression model).

Outcome	Predictor	*p*	OR	95% CI
Shockable Initial Rhythm	Population > 100,000	0.013	0.543	0.335–0.880
	Female Sex	0.02	0.567	0.352–0.914
	Cardiac Cause	0.002	3.767	1.618–8.770
	Out-of-Residence Location	<0.001	2.891	1.801–4.641
	Bystander Full CPR (vs. CCO)	<0.001	2.272	1.449–3.564
Any ROSC	Patient Age ≤ 65 years	0.006	1.803	1.187–2.737
	Cardiac Cause	<0.001	0.340	0.192–0.601
	Out-of-Residence Location	<0.001	2.554	1.617–4.032
	DA (vs. non-DA) Bystander CPR	0.046	0.648	0.423–0.992
	Bystander Full CPR (vs. CCO)	0.025	1.607	1.061–2.432
	Shockable Initial Rhythm	<0.001	5.461	4.647–6.418
Admission ROSC	Population > 100,000	<0.001	0.468	0.301–0.729
	Cardiac Cause	0.004	0.486	0.299–0.791
	Out-of-Residence Location	0.044	1.585	1.013–2.480
	Shockable Initial Rhythm	<0.001	4.434	3.639–5.404

Legend: OR—odds ratio; CI—confidence interval; CPR—cardiopulmonary resuscitation; CCO—cardiac compressions only; DA—dispatcher-assisted; ROSC—return of spontaneous circulation.

**Table 5:** Hospitalization duration in patients with different 5-min time intervals between emergency call and return of spontaneous circulation as well as emergency medical service arrival at scene and return of spontaneous circulation.

Emergency Call to Any ROSC Time Interval (min)	Maximum Duration of Hospitalization (days)	Med (IQR) Days in Survived Patients	Med (IQR) Days in All Patients
0–5	10	9 (9–9)	9 (7–9)
6–10	39	16 (10–16)	10 (3–16)
11–15	30	9 (4–30)	5 (1–15)
>15	62	10 (5–23)	4 (1–21)
**EMS scene arrival to any ROSC time interval (min)**	**Maximum duration of hospitalization (days)**	**Med (IQR) days in survived patients**	**Med (IQR) days in all patients**
0–5	10	9 (9–10)	4 (1–6)
6–10	39	16 (7–30)	1 (1–8)
11–15	20	5 (5–20)	1 (0–4)
>15	62	18 (5–25)	3 (1–31)

Legend: ROSC—return of spontaneous circulation; IQR—interquartile range; EMS—emergency medical service.

## Data Availability

No new data were created or analyzed in this study. Data sharing is not applicable to this article.
